# Carbazole-based aggregation-induced phosphorescent emission-active gold(I) complexes with various phosphorescent mechanochromisms

**DOI:** 10.3389/fchem.2022.1083757

**Published:** 2022-12-01

**Authors:** Zhao Chen, Xiao-Wen Deng, Xiao-Yan Wang, An-Qi Wang, Wen-Tao Luo

**Affiliations:** ^1^ Jiangxi Key Laboratory of Organic Chemistry, Jiangxi Science and Technology Normal University, Nanchang, China; ^2^ College of Chemical Engineering, Shijiazhuang University, Shijiazhuang, China; ^3^ School of Chemistry and Chemical Engineering, Jiangxi Science and Technology Normal University, Nanchang, China

**Keywords:** carbazole, Gold(I) complexes, different substituents, aggregation-induced phosphorescent emission, mechano-responsive phosphorescence

## Abstract

A series of carbazole-containing gold(I) complexes modified with different substituents were successfully designed and synthesized, and their molecular structures were characterized by nuclear magnetic resonance spectroscopy and mass spectrometry. The aggregation-induced behaviors of these gold(I) complexes were studied by ultraviolet/visible and photoluminescence spectroscopy. Meanwhile, their mechanical force-responsive emissive properties were also investigated *via* solid-state photoluminescence spectroscopy. Interestingly, all these gold(I)-based luminogenic molecules were capable of exhibiting aggregation-induced phosphorescent emission phenomena. Furthermore, their solids of three gold(I) complexes displayed contrasting mechano-responsive phosphorescence features. More specifically, trifluoromethyl or methoxyl-substituted luminophores **1** and **3** demonstrated mechanochromic behaviors involving blue-shifted phosphorescence changes, and their mechanoluminochromic phenomena were reversible. However, the solid-state phosphorescence of phenyl-substituted luminophor **2** was not sensitive to external mechanical force.

## Graphical Abstract

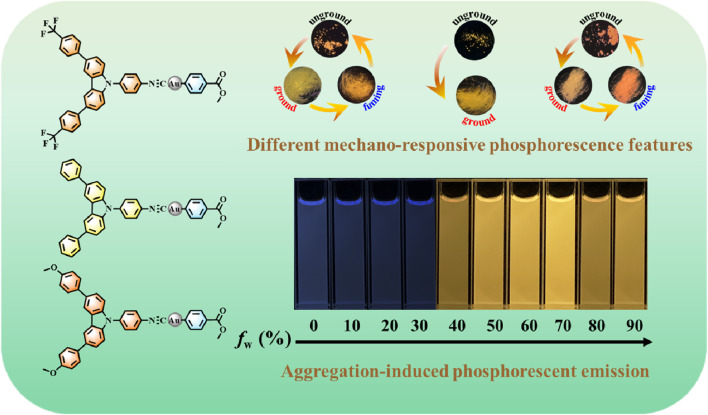

## Introduction

**FIGURE 1:**
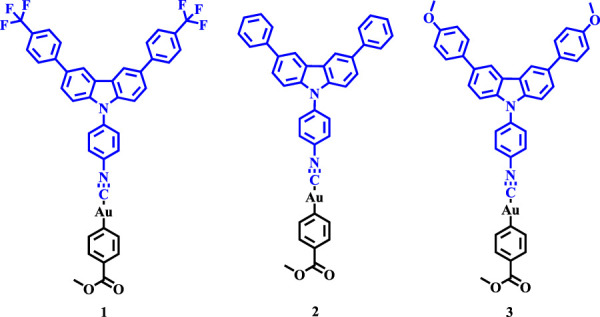
Molecular structures of gold(I) complexes **1**-**3**.

For more than a decade, investigators in chemistry, biology, and materials science have paid more and more attention to construct photochemical/physical materials for highly efficient utilization of lights in various fields, such as solar cells, fluorescent switches, data storage, bio-applications, and photodynamic/thermal therapy (Ni et al., 2018; Ye et al., 2019; Lv et al., 2020). In particular, mechanochromic (MC) luminescence materials that display a solid-state luminous color change upon external force stimulus are a class of important functionalized smart materials, and MC metal complexes are promising candidates of this type of materials. Two key factors for developing high-efficiency MC materials are bright aggregative-state luminescence and high color contrast before and after grinding (Zhang J. et al., 2021; Yang W. et al., 2020; Dong et al., 2020; Ding et al., 2013; Wang et al., 2022). However, their light emissions of traditional luminescent dyes dramatically decrease upon aggregation due to the notorious aggregation-caused quenching (ACQ) effect, which largely limits the effective applications of emissive materials (Leung et al., 2013; Xue et al., 2016; Shi et al., 2018; Huang G. et al., 2019; Echeverri et al., 2020; Liu et al., 2021; Ge et al., 2022). Fortunately, in 2001, Tang et al. introduced an intriguing phenomenon that was known as aggregation-induced emission (AIE) (Mei et al., 2015; Chen et al., 2016; Qian and Tang, 2017; Kunitski et al., 2019; Yang et al., 2020b; Yin et al., 2021a; Sun et al., 2021). Subsequently, Park et al. reported aggregation-induced emission enhancement (AIEE) effect in 2012. Remarkably, luminogens with AIE or AIEE properties possess anti-ACQ characteristics (Dong et al., 2009; An et al., 2012; Gopikrishna et al., 2018). In the recent twenty years, AIE or AIEE luminogenic molecules have been proved to be valuable emitters for exploiting high-performance stimuli-responsive luminescent materials (Wu et al., 2021). Up till now, the majority of molecules simultaneously displaying AIE and mechanoluminochromic behaviors have been reported to be pure organic compounds (Tong et al., 2014; Li W. et al., 2017; Huang L. L. et al., 2019; Yin et al., 2021b; Zhang X. et al., 2021). In contrast, the number of metal-organic complexes possessing these noteworthy properties remains inadequate. In comparison with organic luminescent compounds, metal-containing complexes are more conducive to realizing phosphorescent emission (Zhang et al., 2014; Zhang et al., 2022). In the last 20 years, gold(I) complexes have aroused the attention of many investigators because of the presence of the fascinating intramolecular and intermolecular aurophilic interactions. Indeed, some gold(I) complexes with rich photophysical natures have been discovered. For instance, in 2008, Ito et al. reported the first example of gold(I) complex possessing reversible MC luminescence behavior (Ito et al., 2008). In 2014, Chen et al. developed the first example of gold(I) complex with aggregation-induced white light-emitting feature (Chen et al., 2014). Next, in 2015, Chen et al. also reported the first example of AIE-active gold(I) complex with crystallization-induced emission enhancement and reversible mechanochromic behaviors (Chen et al., 2015). In 2019, Wang et al. discovered the first example of an excitation wavelength-dependent nearly pure white-emissive gold(I)-containing crystal material (Wang et al., 2019). More importantly, gold(I) complexes are capable of emitting long-lived room-temperature phosphorescence, which is very significative for the exploitation of high-performance MC light-emitting materials (Chen et al., 2017). In this work, we described three novel carbazole-modified mononuclear gold(I) complexes with different substituents involving trifluoromethylphenyl, phenyl and methoxyphenyl (Figure 1), and all the three gold(I) complexes showed aggregation-induced phosphorescent emission behaviors with long lifetimes in the microsecond range in aggregated states, which were longer than the lifetimes of most pure organic AIEgens. Additionally, most luminescent mechanochromic compounds showed red-shifted emission upon application of a mechanical force, and there have only been a few reports of materials exhibiting blue-shifted emission upon mechanical stimulation. It is worth noting that the trifluoromethyl or methoxyl-substituted mononuclear gold(I) complexes **1** and **3** showed hypsochromic MC phosphorescence phenomena, while no MC phenomenon was observed for phenyl-substituted mononuclear gold(I) complex **2**.

## Materials and methods

### Experimental

#### General

All reagent were carried out by using standard Schlenk techniques under an argon atmosphere, unless otherwise stated. CH_2_Cl_2_ was dried with CaH_2_ then distilled. THF was dried with Na and then distilled. All starting materials were obtained commercially as analytical-grade and used without further purification. Using tetramethylsilane (TMS) as an internal standard, ^1^H NMR (500 MHz) spectra were measured on American Varian Mercury Plus 500 spectrometer (500 MHz) in deuterated solvents and analyzed at room temperature using the Bruker NMR software package-TopSpin. ^1^H NMR chemical shifts were referenced to the residual solvent signal (7.26 ppm for CDCl_3_), and ^19^F NMR chemical shifts are relative to C_6_F_6_ (*δ* = −163.00). Mass spectra (MS) were recorded by Bruker AmaZon SL Ion Trap Mass spectrometer. Fluorescence spectra were conducted by Hitachi F-4600 fluorescence spectrophotometer and the Edinburgh FLS1000 instrument with an integrating sphere. The UV-vis absorption spectra were obtained on an Agilent 8454 UV-Vis spectrophotometer. The X-ray diffraction (XRD) patterns of complexes 1 and 3 in different solid states were recorded by Shimadzu XRD-6000 diffractometer using Ni-filtered and graphite-monochromated Cu Kα radiation (*λ* = 1.54 Å, 40 kV, 30 mA). Dynamic light scattering (DLS) measurements were characterized by NanoBrook 90 Plus (Brookhaven Instruments). OriginLab OriginPro 9.0 software package was used for spectral processing. All reactions using monitored by precoated TLC plates under UV light at 254 nm.

### Synthesis

#### Synthesis of monoisonitrile ligands **I-3**, **II-3** and **III-3**

Monoisonitrile ligands **I-3**, **II-3** and **III-3** were synthesized in a similar manner (Zhao et al., 2019). The synthesis of compound **I-3** was taken as an example: the mixture of compound **I-1** (5.46 g, 10 mmol) and formic acid (80 ml) was stirred overnight at 110°C. After completion of the reaction, formic acid was removed from the reaction system by distillation, and the residual mixture was extracted with methylene chloride (5 × 60 ml). The organic layer was washed with brine, dried over Na_2_SO_4_, and concentrated in vacuum to give the product **I-2** (white solid). Next, the corresponding CH_2_Cl_2_ solution (50 ml) of **I-2** and triethylamine (5 ml) was cooled to 0°C. Triphosgene (3.26 g, 11 mmol) of CH_2_Cl_2_ solution (30 ml) was added to the mixture drop by drop and then refluxed for 3 h. After the reaction was completed, the mixture was cooled to room temperature. 10% Na_2_CO_3_ solution (80 ml) was dropped to the system for neutralization. The mixture was extracted with trichloromethane (3 × 50 ml), the organic layer was washed with brine, dried over Na_2_SO_4_, and concentrated in vacuum. The residue was purified by column chromatography and the expected white isonitrile ligand **I-3** was obtained in 70% yield.

#### Synthesis of gold(I) chloride complexes **I-4**, **II-4** and **III-4**

The synthesis of gold(I) chloride complexes **I-4**, **II-4** and **III-4** were prepared according to the procedure described in reference (Seki et al., 2016).

#### Synthesis of complexes **1**-**3**

**SCHEME 1:**
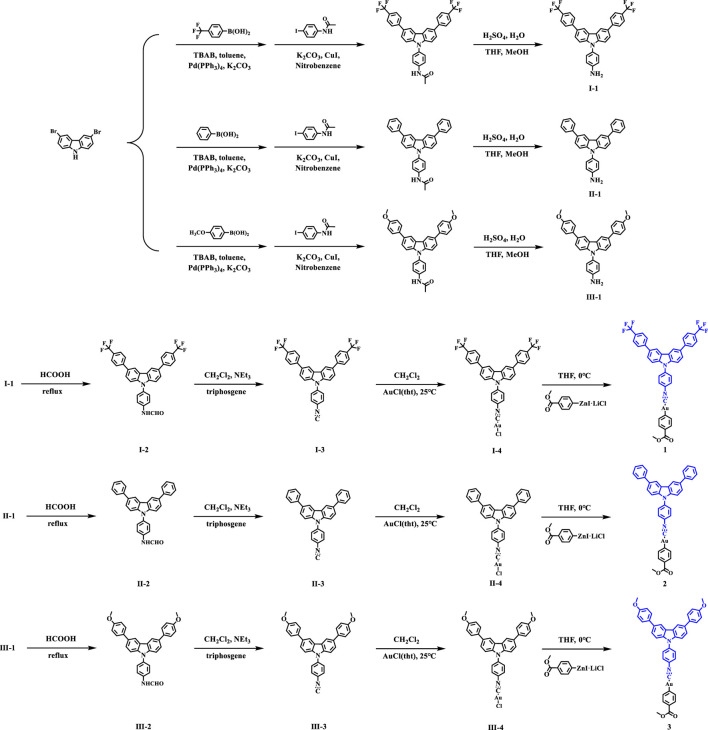
Synthesis routes of compounds **1**-**3**.

As displayed in Scheme 1, the synthesis of complex **1** was taken as an example: gold(I) chloride complex **I-4** (753.1 mg, 1 mmol) was added to a dried reaction flask and dried with three cycles of vacuum/argon. Then, organic zinc reagent (554.7 mg, 1.5 mmol) was dissolved in anhydrous THF (50 ml) at 0°C and stirred for 12 h. At the end of the reaction, the mixture was quenched with phosphate buffer solution (PBS) and extracted three times with CH_2_CH_2_. The organic phase was dried with sodium sulfate, and the solvent was removed *in vacuo*. Finally, the expected white solid products **1** was obtained by flash column chromatography with yield of 65%. **1**: ^1^H NMR (500 MHz, CDCl_3_) *δ* 8.43 (s, 2H), 8.14 (d, *J* = 10.0 Hz, 1H), 7.93 (d, *J* = 5.0 Hz, 2H), 7.82 (s, 6H), 7.76 (m, *J* = 5.0, 6H), 7.58–7.54 (m, 4H), 3.95 (s, 1H), 3.88 (s, 3H). ESI-MS (m/z): ESI-MS (m/z): [M + H]^+^ of C_41_H_25_AuF_6_N_2_O_2_: 889.1564 (calcd), 889.1570 (found). **2**: Yield: 71%. ^1^H NMR (500 MHz, CDCl_3_) *δ* 8.40 (s, 2H), 7.93 (d, *J* = 5.0 Hz, 2H), 7.83 (m, 4H), 7.72 (t, *J* = 7.5 Hz, 6H), 7.59 (d, *J* = 10.0 Hz, 2H), 7.53–7.49 (m, 6H), 7.38 (t, *J* = 7.5 Hz, 2H), 3.88 (s, 3H). ESI-MS (m/z): [M + H]^+^ of C_39_H_27_AuN_2_O_2_: 753.1816 (calcd), 758.1814 (found). **3**: Yield: 71%. ^1^H NMR (500 MHz, CDCl_3_) *δ* 8.33 (s, 2H), 7.93 (d, *J* = 5.0 Hz, 2H), 7.81 (s, 3H), 7.66 (d, *J* = 5.0 Hz, 7H), 7.58 (d, *J* = 10.0 sHz, 2H), 7.51 (d, *J* = 10.0 Hz, 2H), 7.05 (d, *J* = 10.0 Hz, 4H), 3.89 (s, 9H). ESI-MS (m/z): ESI-MS (m/z): [M + H]^+^ of C_41_H_31_AuN_2_O_4_: 813.2028 (calcd), 813.2027 (found).

## Results and discussion

### Synthesis

The monoisonitrile ligands **I-3**, **II-3** and **III-3** were prepared according to the procedure described in reference (Seki et al., 2016). The complexes **1-3** were obtained in high yields and the strategies were presented in Scheme 1 *.*

### Aggregation-induced phosphorescent emission properties of complexes **1-3**

In order to survey the aggregation-induced emission properties of complexes **1-3**, the UV-vis absorption spectra with different water fractions (*f*_W_) were investigated in N, N-dimethylformamide (DMF)-H_2_O mixtures (Osawa et al., 2010; Li Y. et al., 2017). The results showed that the level-off tails appeared in the visible region with increasing *f*_W_, which could be attributed to the well-known Mie scattering effect and indicated the formation of nano-aggregates at the same time (Supplementary Figure S1).

**FIGURE 2:**
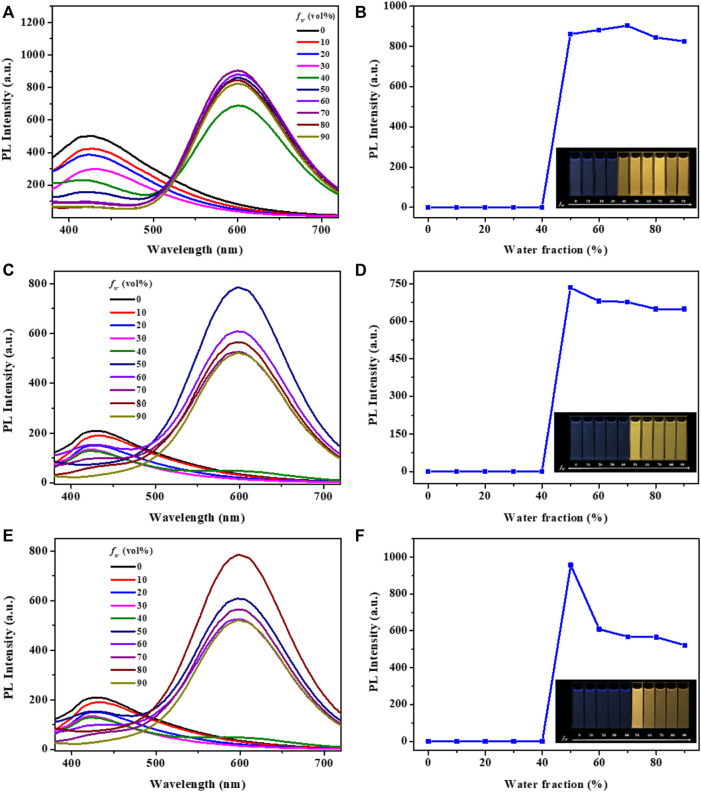
Fluorescence spectra of the complexes **1 (A)**, **2 (C)**, **3 (E)** in DMF-H_2_O mixtures with various water fractions. Changes in emission intensity of complexes **1 (B)**, **2 (D)**, **3 (F)** in DMF-H_2_O mixtures with various water fractions. Inset: Fluorescence images of complexes **1 (B)**, **2 (D)**, **3 (F)** in different DMF-H_2_O mixtures under 365 nm UV lamp (c: 2.0 × 10^–5^ mol L^−1^).

**FIGURE 3:**
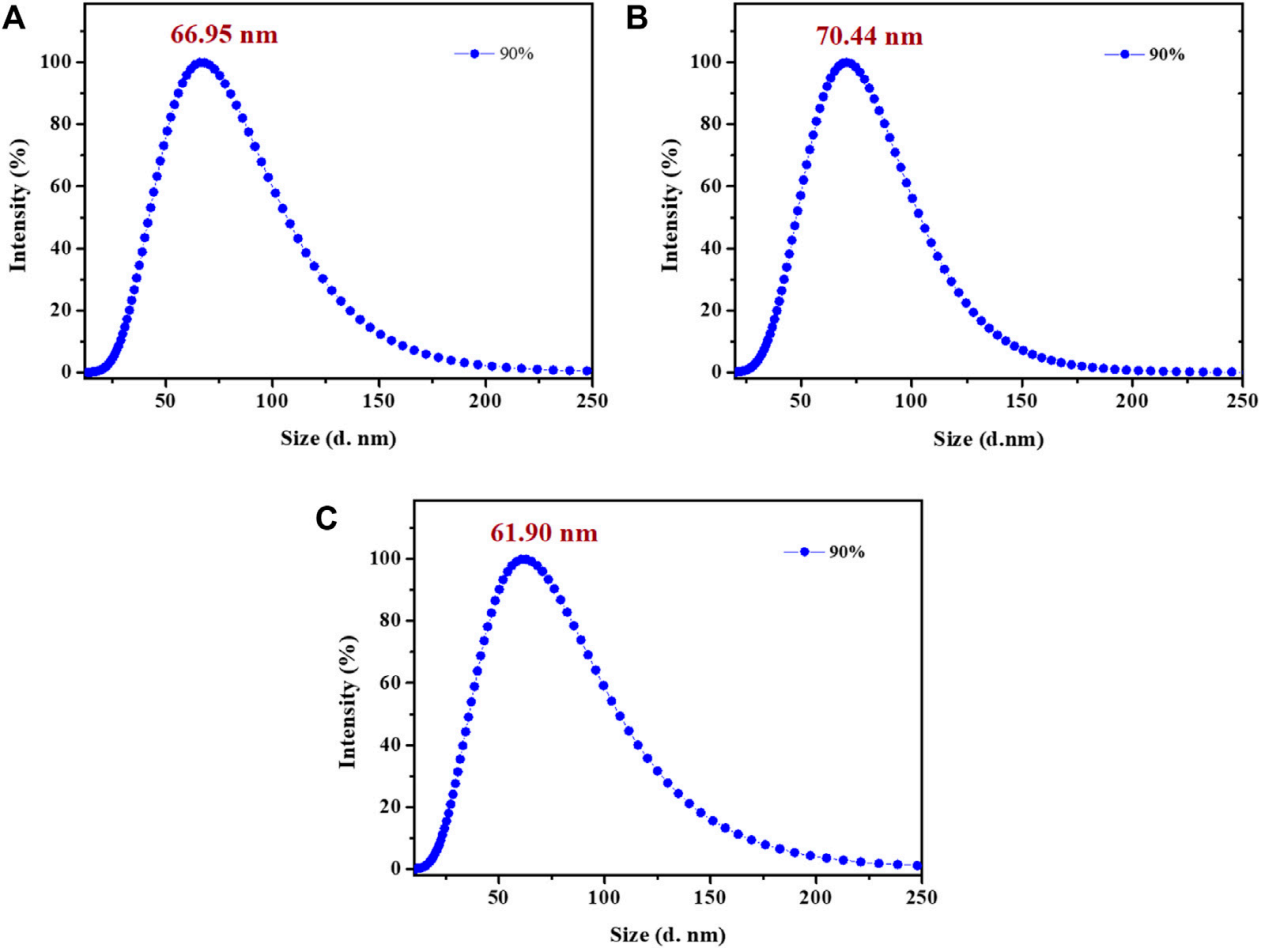
Size distribution curves of **1 (A)**, **2 (B)** and **3 (C)** in DMF-water mixtures with 90% volume fraction of water (*E*_x_: 365 nm; *c*: 2.0 × 10^–5^ mol L^−1^).

Subsequently, the photoluminescence spectra of complexes **1-3** in DMF-H_2_O mixtures with varying water fractions were investigated. The solution of these complexes in pure DMF showed broad and Gaussian shaped emission peaks at around 420 nm, which might be originated from charge transfer (CT) states, rather than ligand-localized transitions. As shown in Figure 2, complex **1** emitted very weak blue emission in pure DMF under UV light excited at 365 nm (Hunks et al., 2000; Ishi-I et al., 2019). However, when the *f*_W_ reached 40%, a new broad emission peak was detected at around 600 nm and the mixture showed obvious yellow emission. As the water fractions continuously increased to 70%, the emission intensity of complex **1** was also enhanced significantly. Clearly, the bright yellow emission of complex **1** could be attributed to the formation of nano-aggregates, which was further confirmed by dynamic light scattering (DLS) measurements in DMF-water mixtures with 90% volume fraction of water (Figure 3). Besides, the formation of aurophilic interactions after aggregating was also responsible for the yellow emission of complex **1**. Decay curve gave its phosphorescence lifetime values as 0.97 μs in aggregated state (Supplementary Figure S2). These results indicated the aggregation-induced phosphorescent emission (AIPE) characteristic of complex **1**. Similarly, bright yellow emissions could also be found until the water fractions increased to 50% for complexes **2** and **3**. The microsecond lifetimes (1.03 μs for **2**; 0.91 μs for **3**) in aggregation proved their excellent AIPE properties. In addition, time resolved fluorescence data of **1-3** have been provided and discussed in this manuscript. Taking compound **1** as a representative example, the decay curve gave its phosphorescence lifetime values as 0.72 μs in aggregated state pure DMF at room temperature (quantum yield of 1%). The phosphorescence lifetime values of compound **2** was 0.47 μs (quantum yield of 0.2%). The phosphorescence lifetime values of compound **3** was 0.35 μs (quantum yield of 0.6%) (Supplementary Figure S3).

### Reversible phosphorescent mechanochromisms of complexes **1** and **3**

**FIGURE 4:**
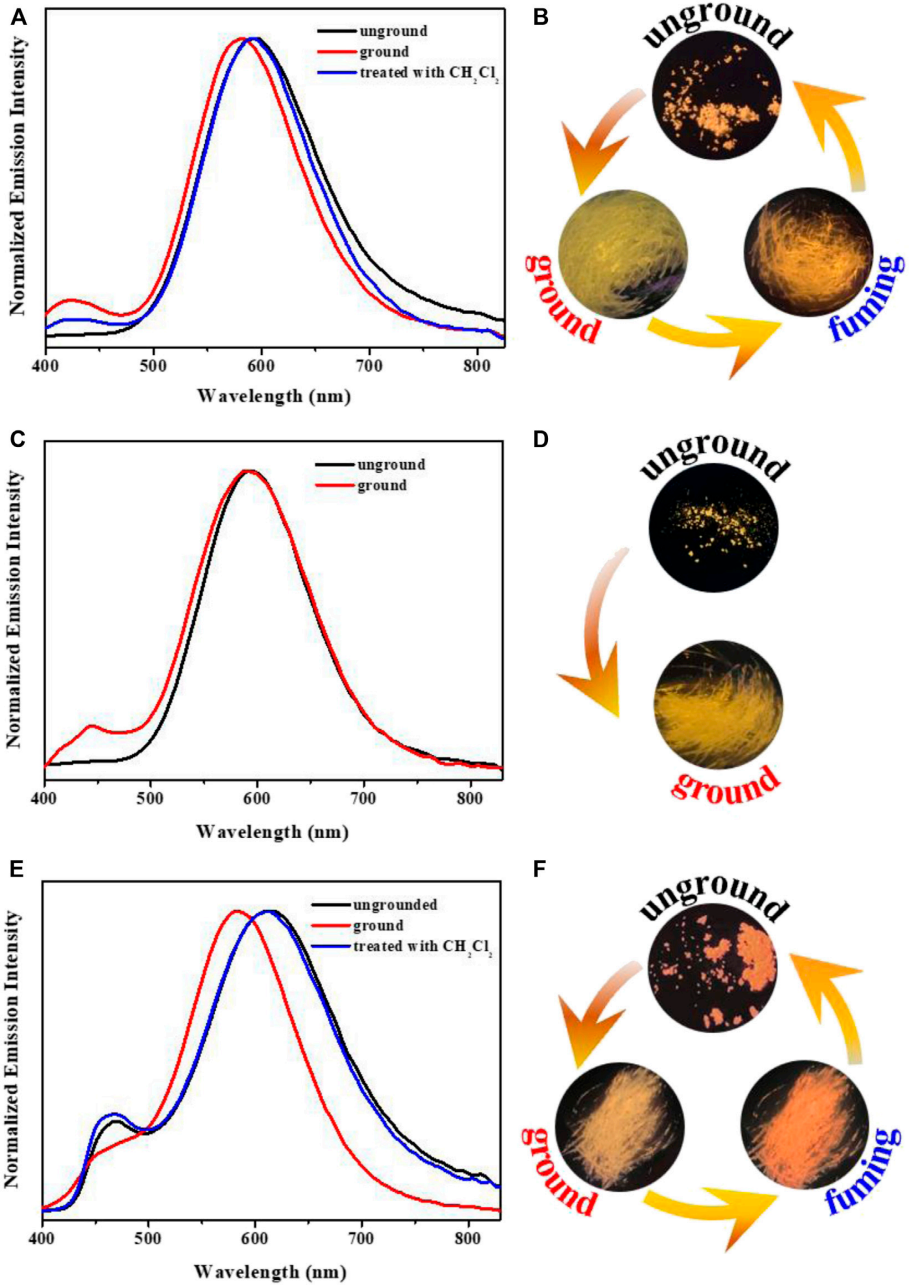
Solid-state PL spectra of complexes **1 (A)**, **2 (C)**, **3 (E)** in various solid states; Fluorescence images of **1 (B)**, **2 (D)**, **3 (F)** in different solid states.

**FIGURE 5:**
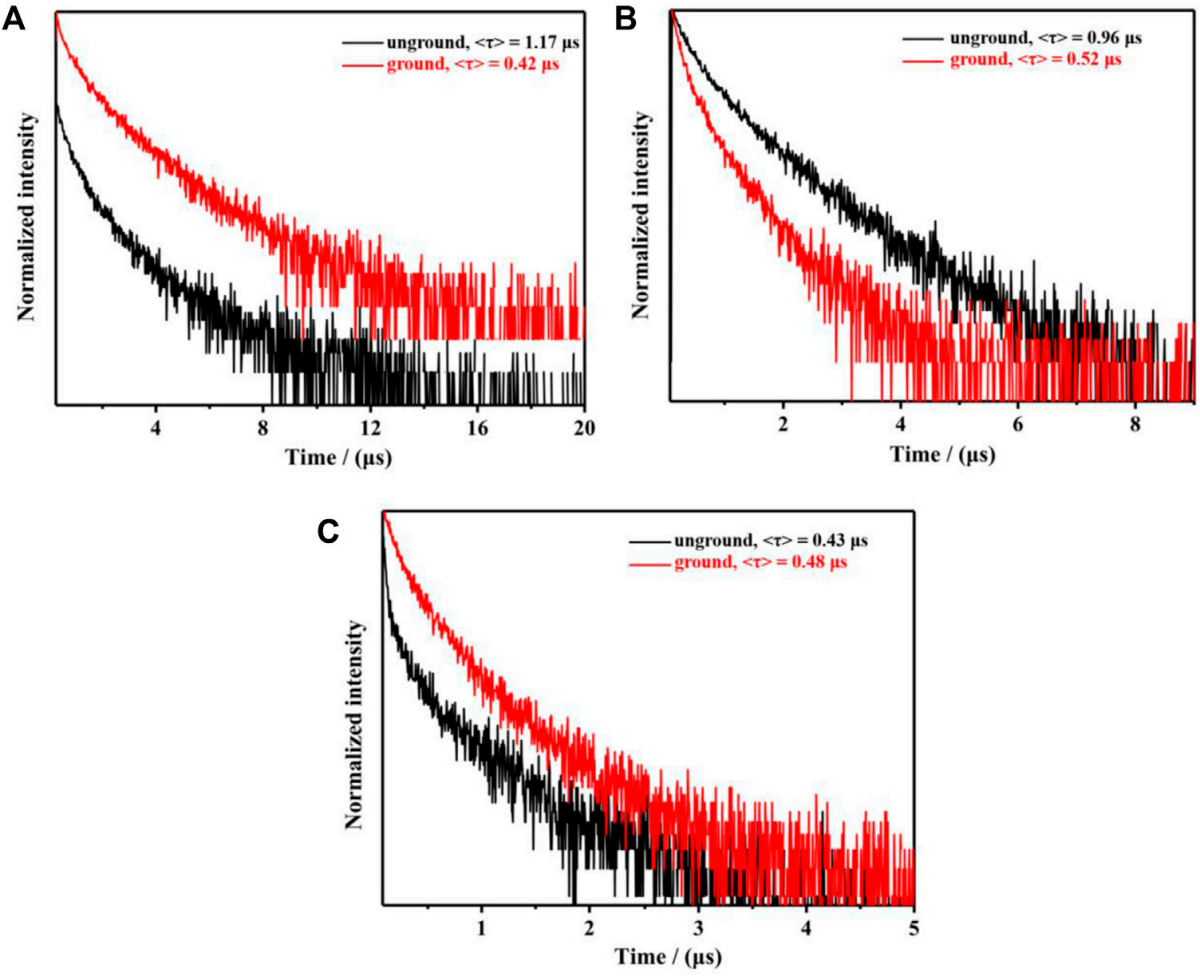
Decay curves of complexes **1 (A)**, **2 (B)**, **3 (C)** in different solid state.

Due to the AIPE characteristics of complexes **1**-**3**, their solids also emitted orange or yellow photoluminescence with the emission peaks at 595 nm, 593 nm and 612 nm, respectively (Figure 4). Their microsecond lifetimes (1.17 μs for **1**; 0.96 μs for **2**; 0.43 μs for **3**) indicated that the emissions of complexes **1**-**3** were originated from phosphorescences (Figure 5). Interestingly, these complexes exhibited various mechano-responsive behaviors (Chi et al., 2012). As displayed in Figure 4, the solid of complex **1** emitted bright orange phosphorescence with the quantum yield of 8%. After grinding, a blue-shifted emission band was observed at 582 nm and the orange color emission was converted to yellow emission. Treating the ground powder with CH_2_Cl_2_ for 30 s, the yellow emission solid returned to its original orange color emission. Furthermore, such mechanical stimulation induced luminescence conversion between orange and yellow could be repeated several times. These experiments indicated that complex **1** exhibited reversible hypsochromic phosphorescence mechanochromism. A similar stimuli-responsive phenomenon occurred in complex **3**. Reversible conversion between orange-red phosphorescence and yellow phosphorescence could be realized by grinding and fuming. Nevertheless, the emission of complex **2** did not change under the mechanical stimulation. This might be due to the unchanged molecular arrangement of the solid **2** after grinding (Yang et al., 2020c; Zhao et al., 2012). What’s more, the absorption spectra of solids **1-3** in various states have been provided (Supplementary Figure S4).

**FIGURE 6:**
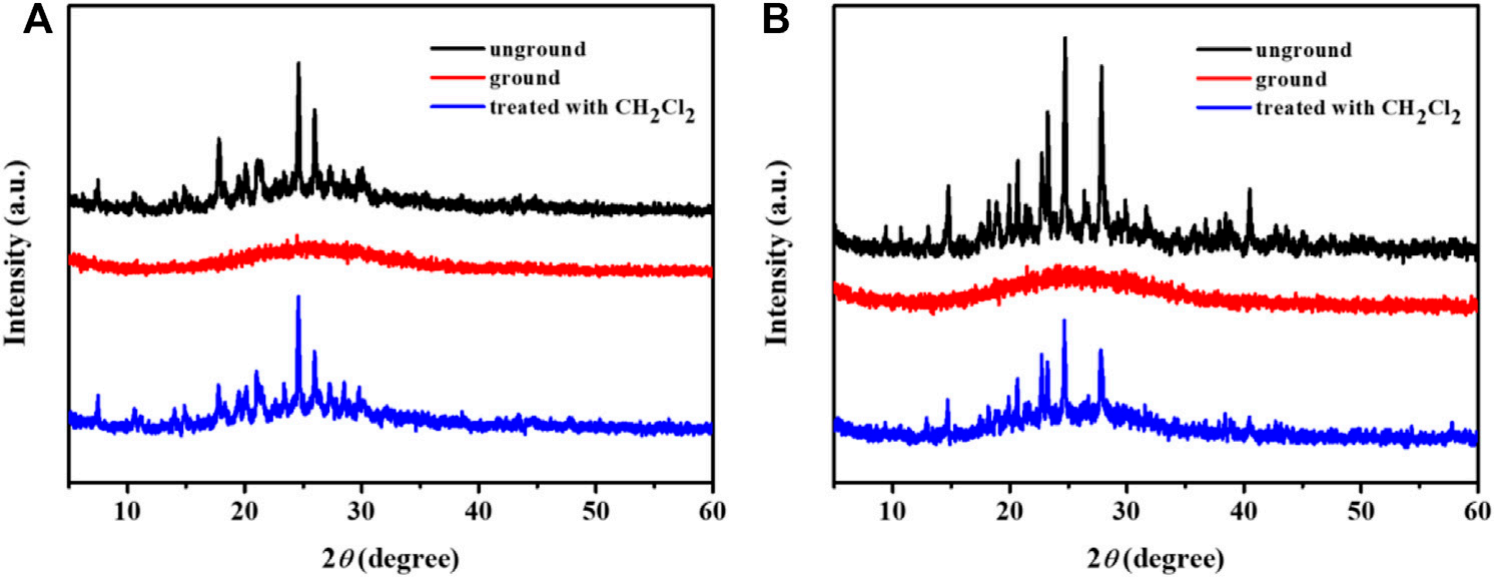
PXRD patterns of **1 (A)** and **3 (B)** in various solid states.

In order to further explore the phosphorescence mechanochromisms of these complexes, the powder X-ray diffraction (PXRD) spectra were carried out. As displayed in Figure 6, the original solid samples of the complex **1** and **3** showed sharp and strong diffraction peaks, indicating the orderly crystalline states. On the contrary, the diffraction peaks decreased and even disappeared after grinding, which mean that the ground states were in amorphous states. As a matter of fact, the original strong diffraction peaks occurred again after fuming the ground solid with CH_2_Cl_2_. Therefore, the PXRD results revealed that the reversible crystalline-amorphous phase transformation was one of the main causes of the reversible hypsochromic phosphorescence mechanochromisms for complexes **1** and **3**.

## Conclusion

In summary, a series of carbazole-containing gold(I) complexes modified with different substituents were successfully designed and synthesized. These novel complexes displayed AIPE behaviors in DMF-water mixtures. We suspected that bright yellow emissions were due to the formation of aurophilic interactions in aggregated states. In solid states, the emission colors of these complexes could be regulated by different substituents. In addition, complexes **1** and **3** exhibited reversible blue-shifted phosphorescence mechanochromisms. The PXRD results revealed that the stimuli-response behaviors were resulted from the crystalline-amorphous phase transformation. This work would be beneficial to design novel AIPE-active metal-organic compounds with remarkable phosphorescent mechanochromisms.

## Data Availability

The original contributions presented in the study are included in the article/Supplementary Material, further inquiries can be directed to the corresponding authors.
