# Supplementary material for: Carbazole-based aggregation-induced phosphorescent emission-active gold(I) complexes with various phosphorescent mechanochromisms
Source: Front Chem. 2022 Dec 1;10:1083757. doi: 10.3389/fchem.2022.1083757 (PMC9752811; doi:10.3389/fchem.2022.1083757)
Supplement: Supplementary file 1 [file DataSheet1.docx]

**Supplementary Information**

**Carbazole-based aggregation-induced phosphorescent emission (AIPE)-active gold(I) complexes with various phosphorescent mechanochromisms**

***Zhao Chen^1*†^, Xiao-wen Deng^1†^, Xiao-Yan Wang^2*^, An-qi Wang^3^ and Wen-Tao Luo^3^***

*^1^ Jiangxi Key Laboratory of Organic* *Chemistry, Jiangxi Science and Technology Normal University, Nanchang 330013, PR China*

*^2^ College of Chemical Engineering, Shijiazhuang University, Shijiazhuang 050035, PR China*

*^3^ School of Chemistry and Chemical Engineering, Jiangxi Science and Technology*

*Normal University, Nanchang 330013, PR China*

*E-mail addresses:* [*chenzhao666@126.com*](mailto:chenzhao666@126.com) *(Z. Chen), wangxiaoyan629@163.com (X.-Y. Wang).*

***^†^*** *These authors have contributed equally to this work.*

**Table of Contents**

**1. FIGURE. S1..........................................................................................................S3**

**2.FIGURE. S2............................................................................................................S4**

**3.FIGURE. S3............................................................................................................S5**

**4.FIGURE. S4............................................................................................................S6**

**5.** **^1^H and ^19^F NMR spectra of 1-3 in CDCl_3_.............................................................S7**

**6. Mass spectra of complexes 1-3 ............................................................................S9**

**1. FIGURE. S1**

**
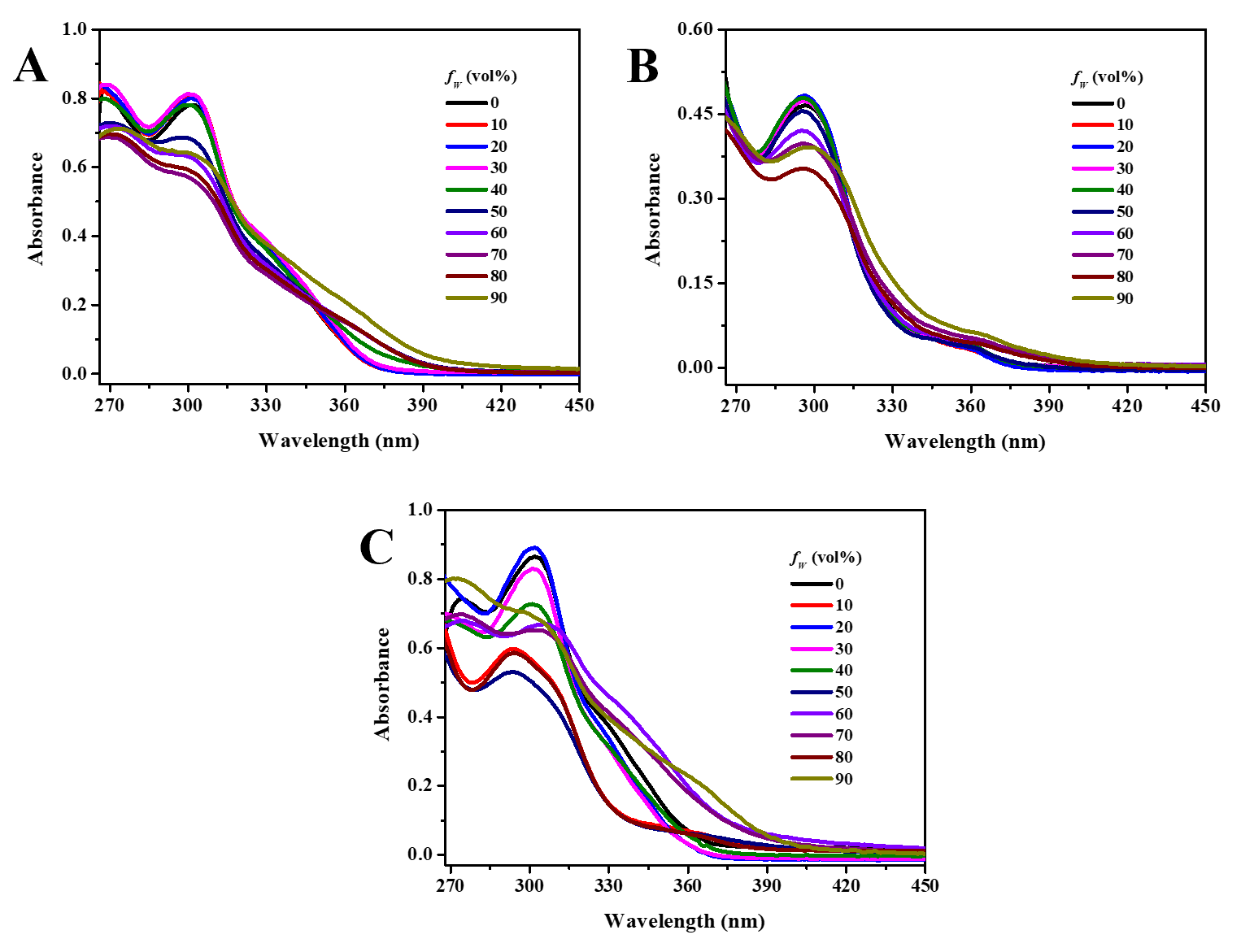
**

**FIGURE. S1 |** UV-vis absorption spectra of compounds **1-3** (A-C) in DMF-H_2_O mixtures (2.0 × 10^-5^ mol L^-1^) with different water fractions (0-90%) at room temperature.

**2. FIGURE. S2**

**
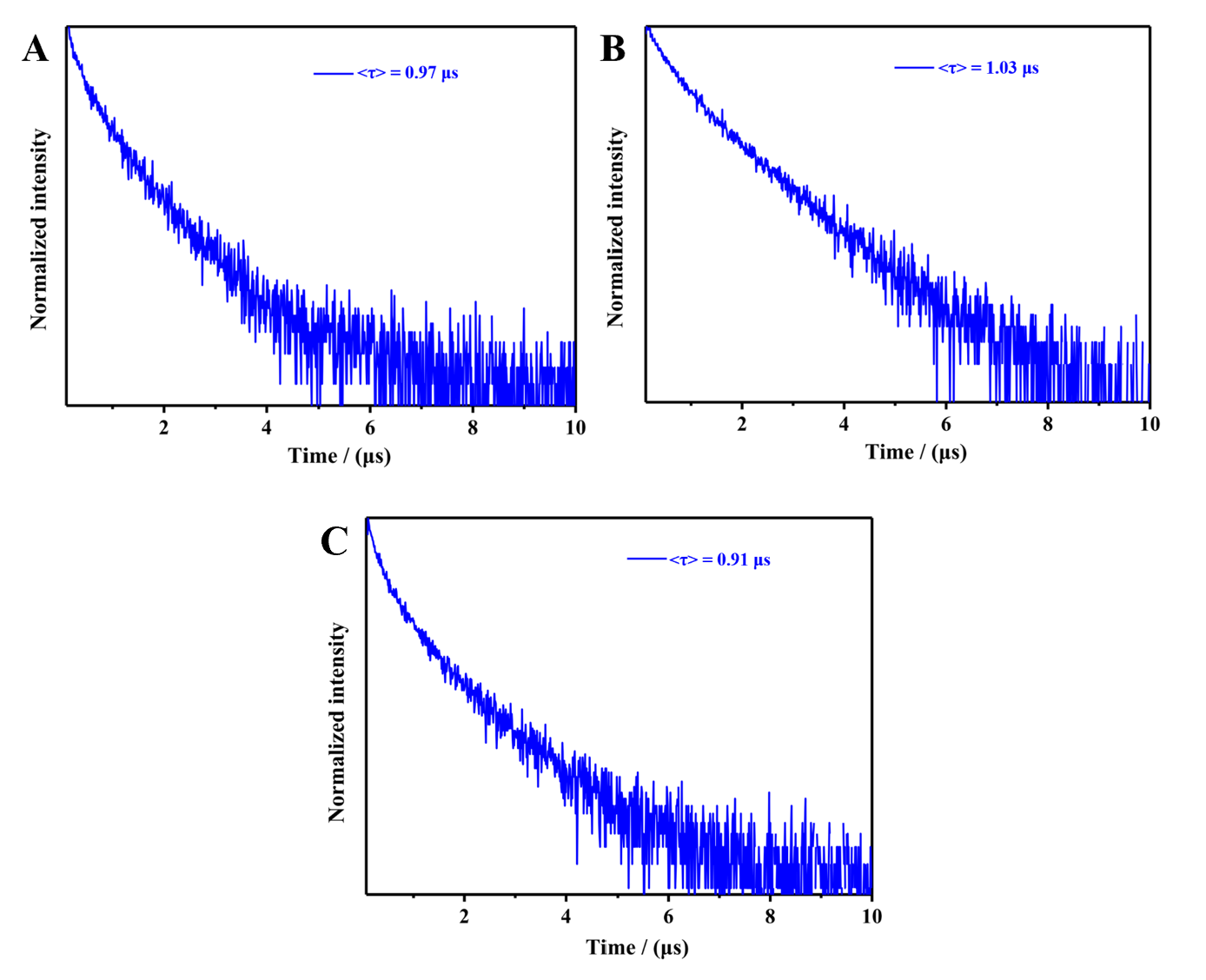
**

**FIGURE. S2 |** Decay curves of complexes **1-3** (A-C) in DMF-H_2_O mixtures (2.0 × 10^-5^ mol L^-1^) with 90% water fraction at room temperature.

**3. FIGURE. S3**

**
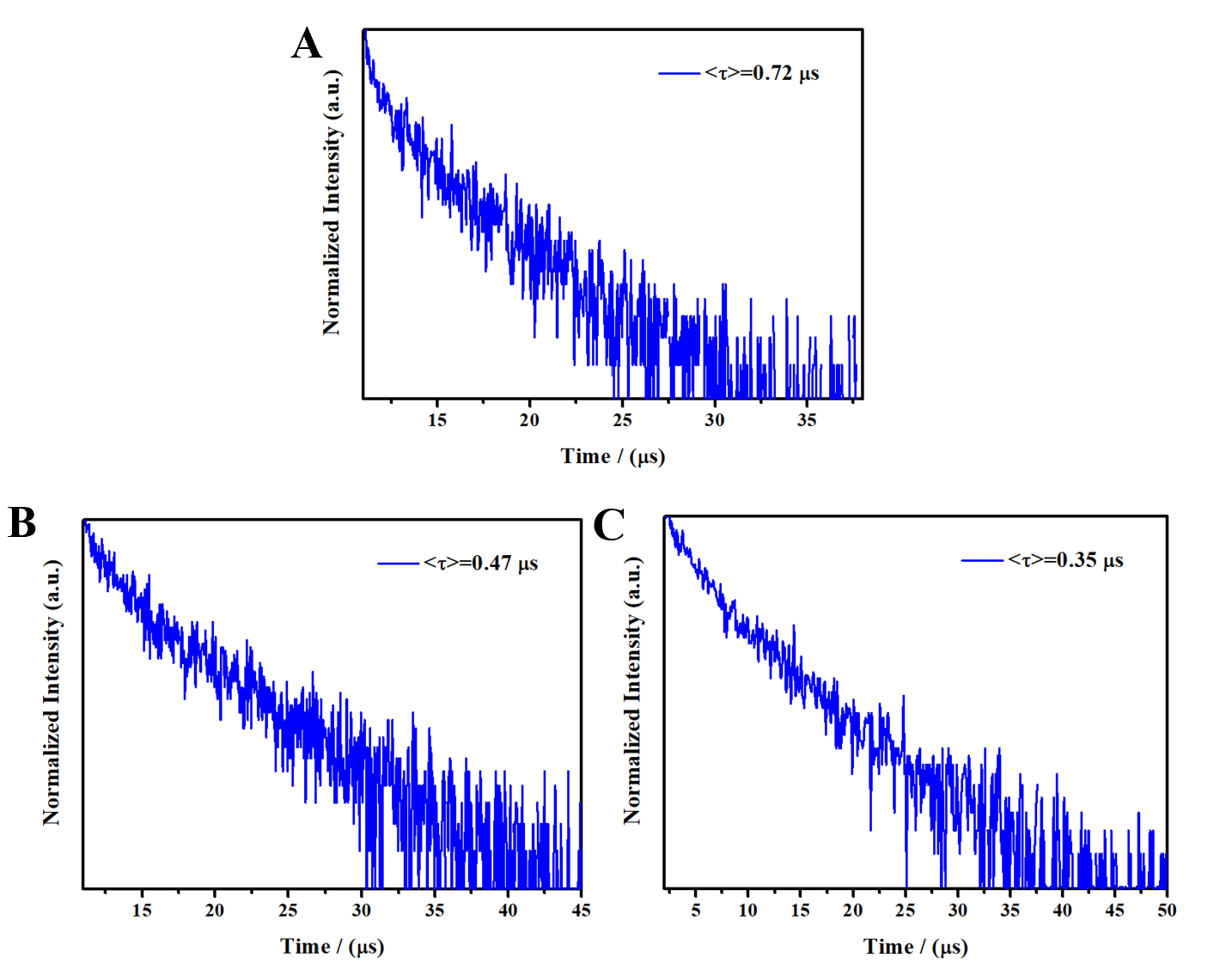
**

**FIGURE. S3 |** Decay curves of complexes **1-3** (A-C) in pure DMF at room temperature.

**4. FIGURE. S4**


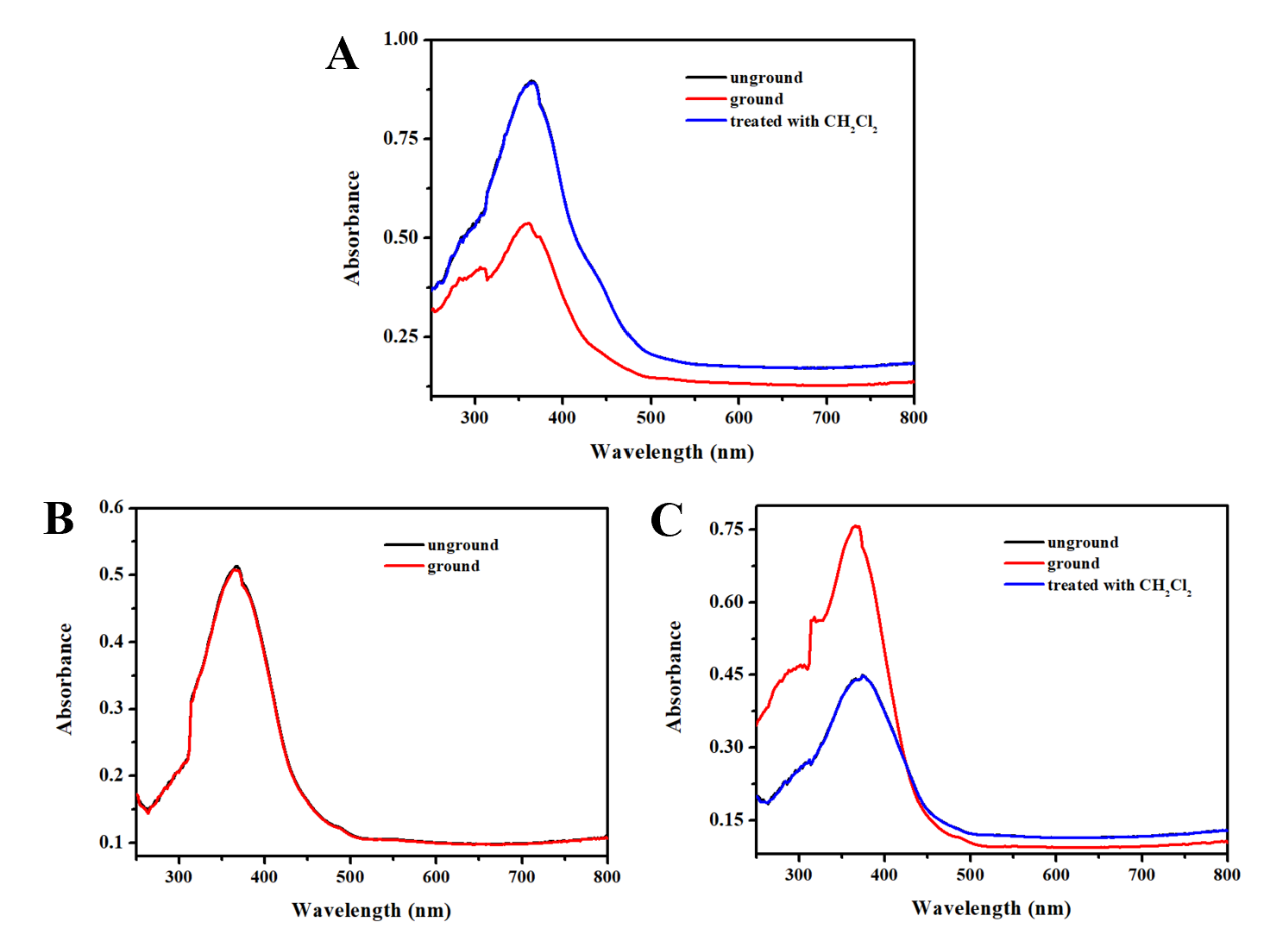


**FIGURE. S4 |** UV-vis absorption spectra of compounds **1-3** (A-C) in in various states.

**5. ^1^H and ^19^FNMR spectra of 1-3 in CDCl_3_**


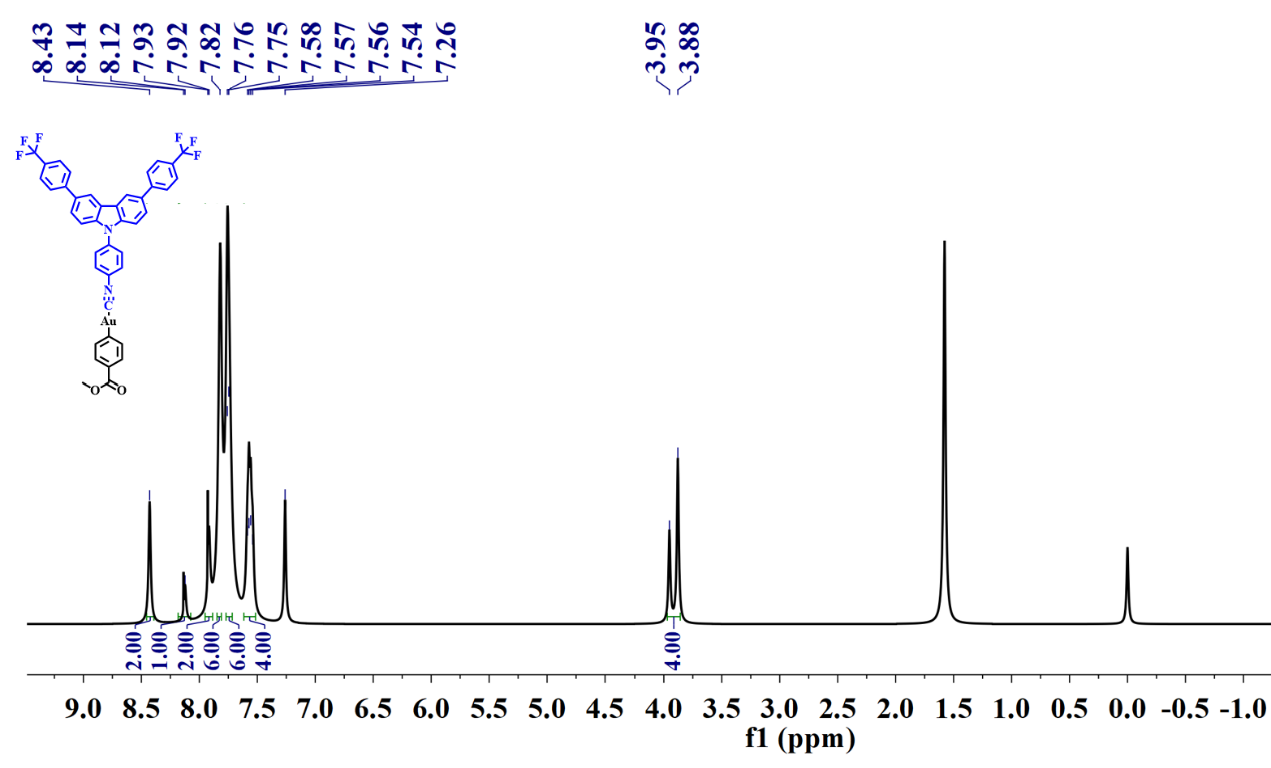

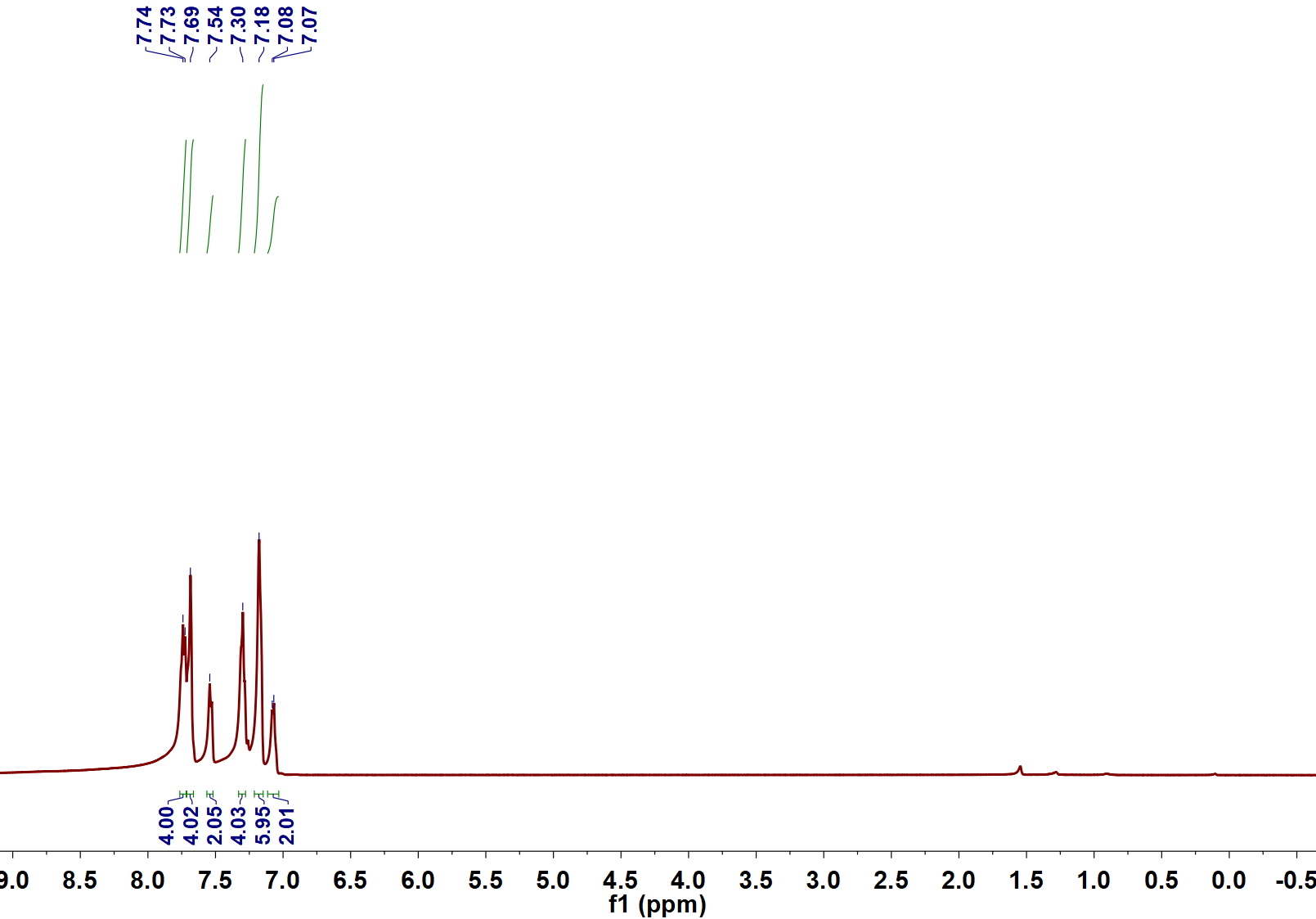

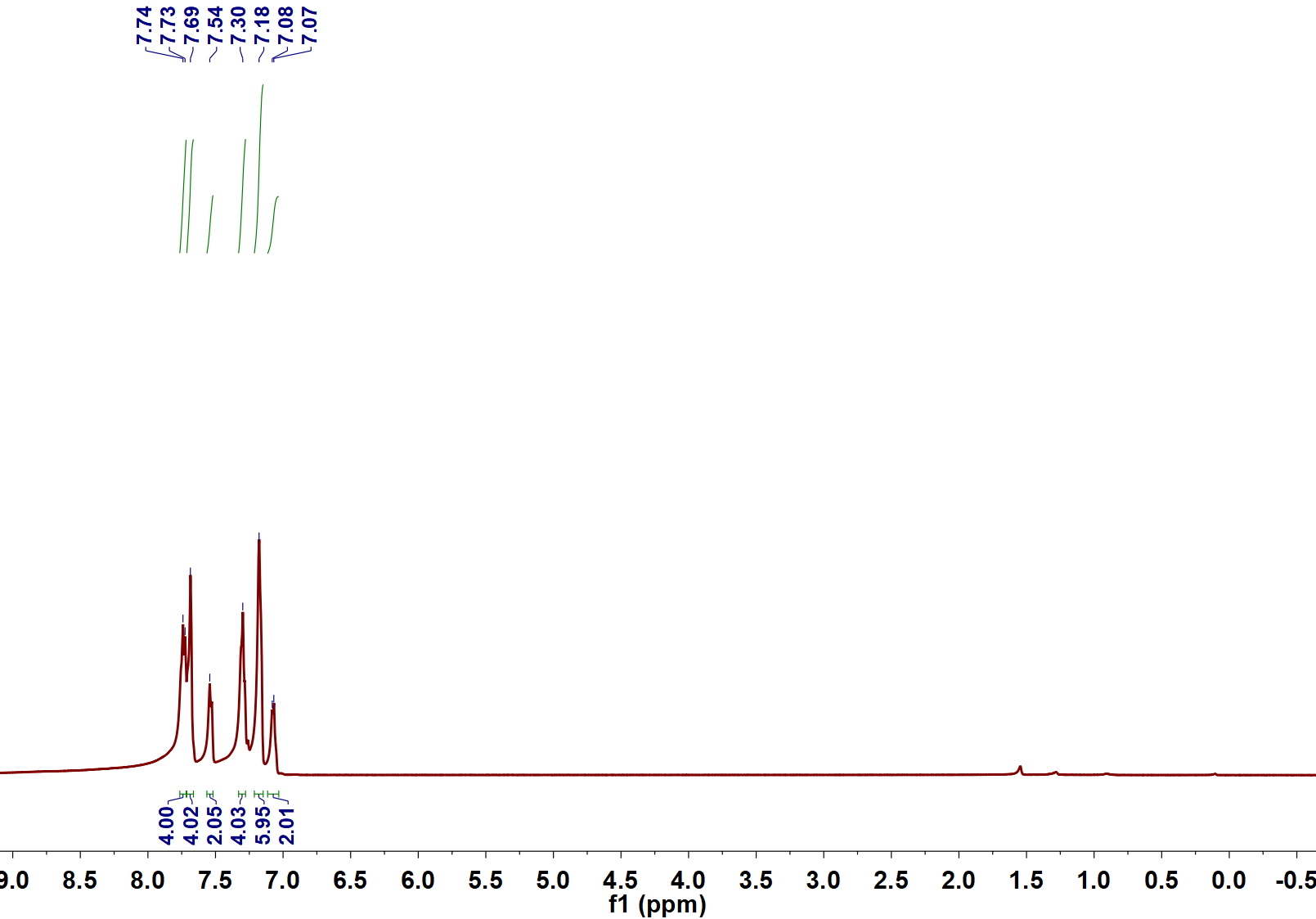


**FIGURE. S5 |** ^1^H NMR spectrum of **1** in CDCl_3_.


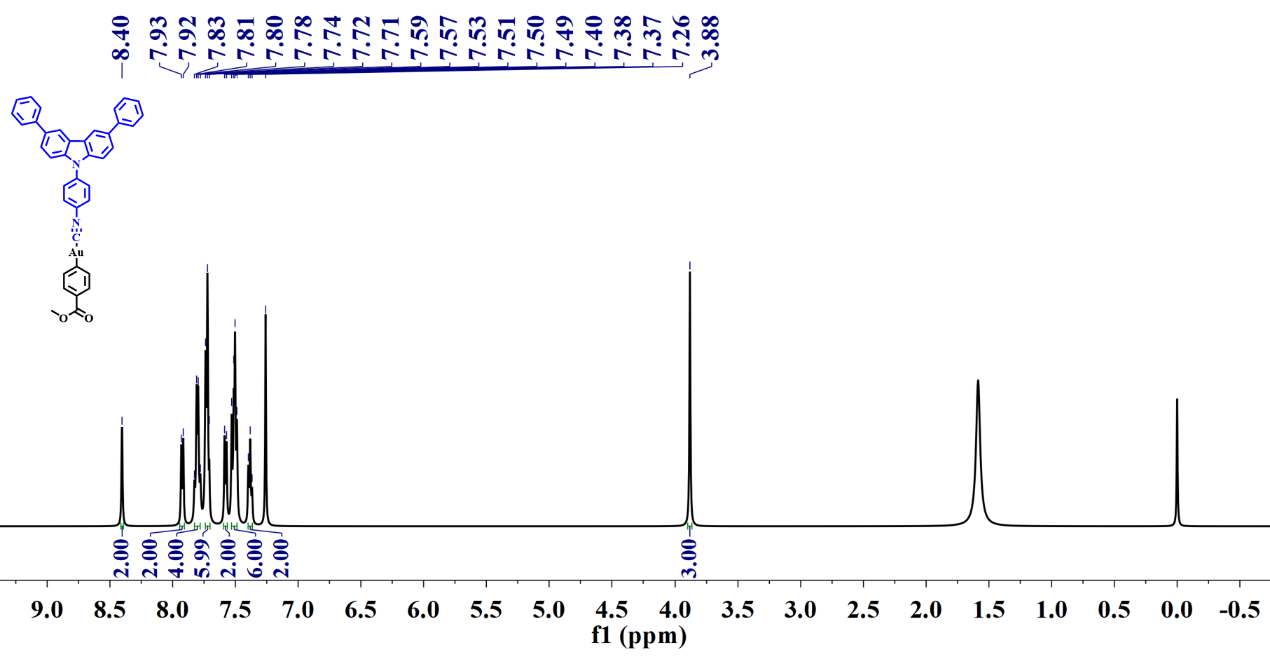


**FIGURE. S6 |** ^1^H NMR spectrum of **2** in CDCl_3_.


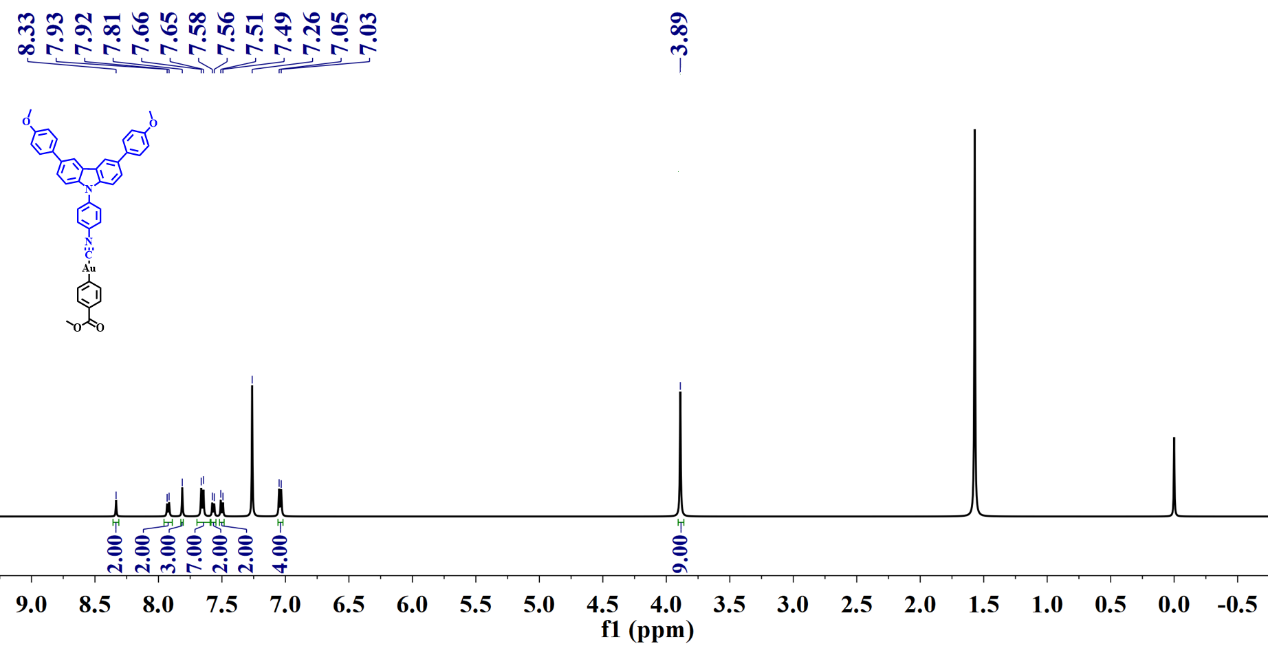


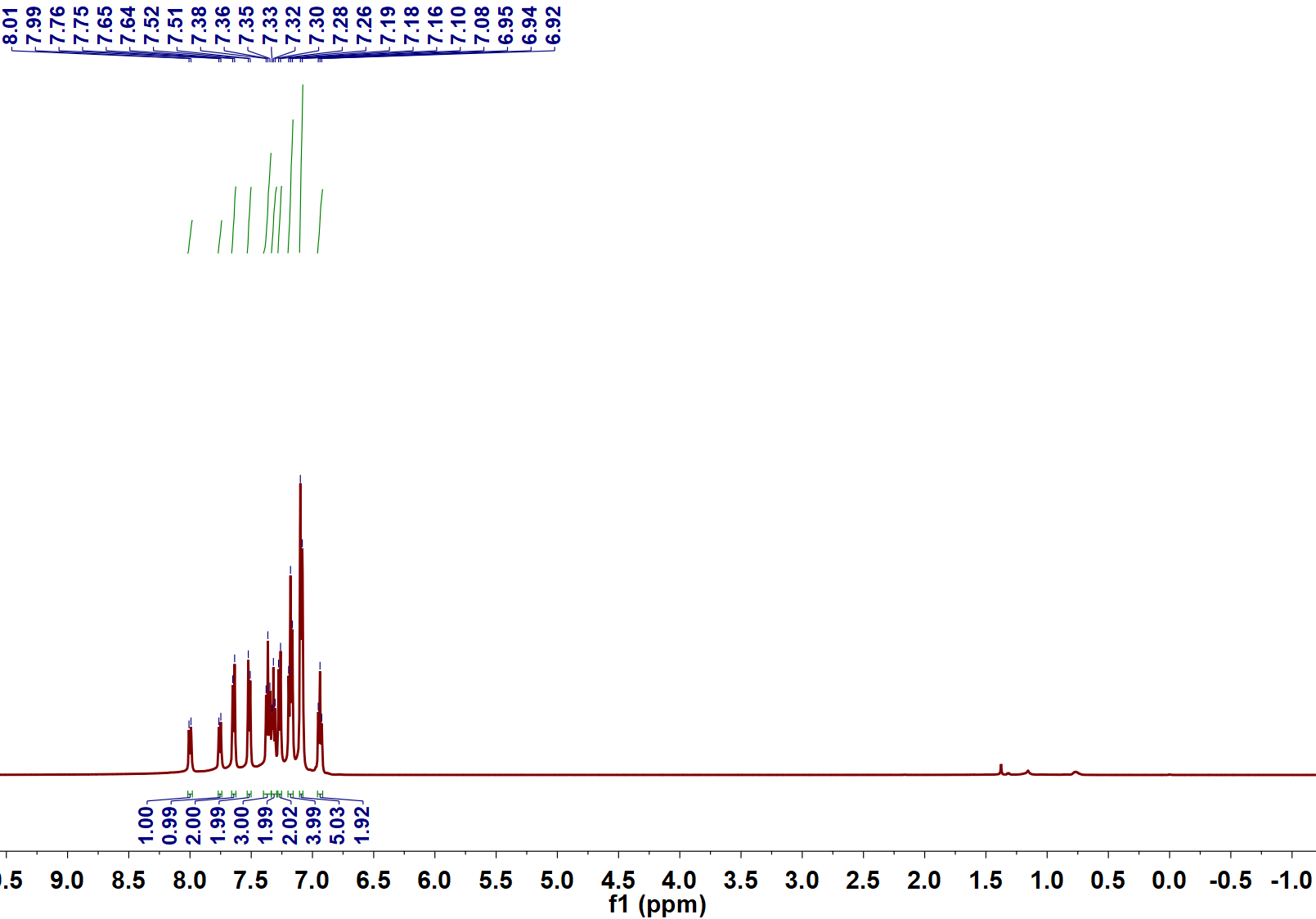
**FIGURE. S7 |** ^1^H NMR spectrum of **3** in CDCl_3_.


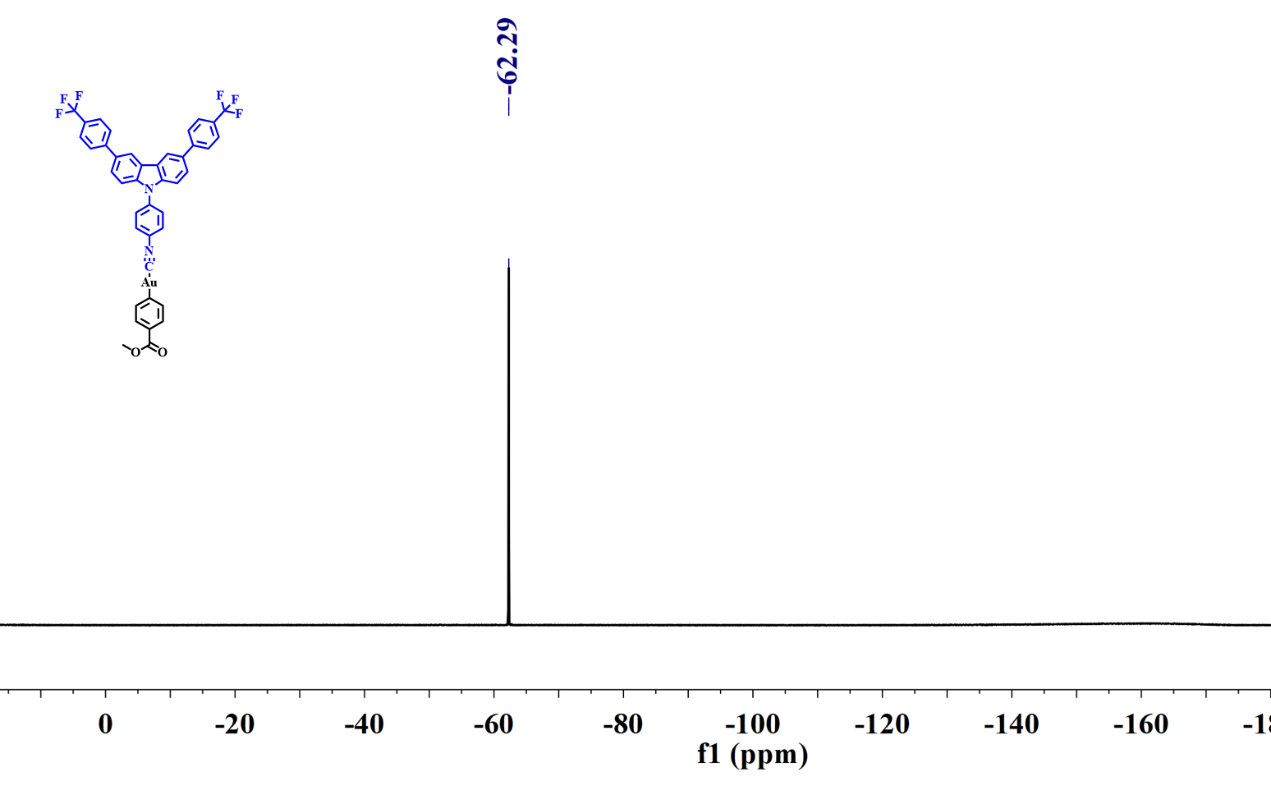


**FIGURE. S8 |** ^19^F NMR spectrum of **1** in CDCl_3_.

**4. Mass spectra of 1-3**

**
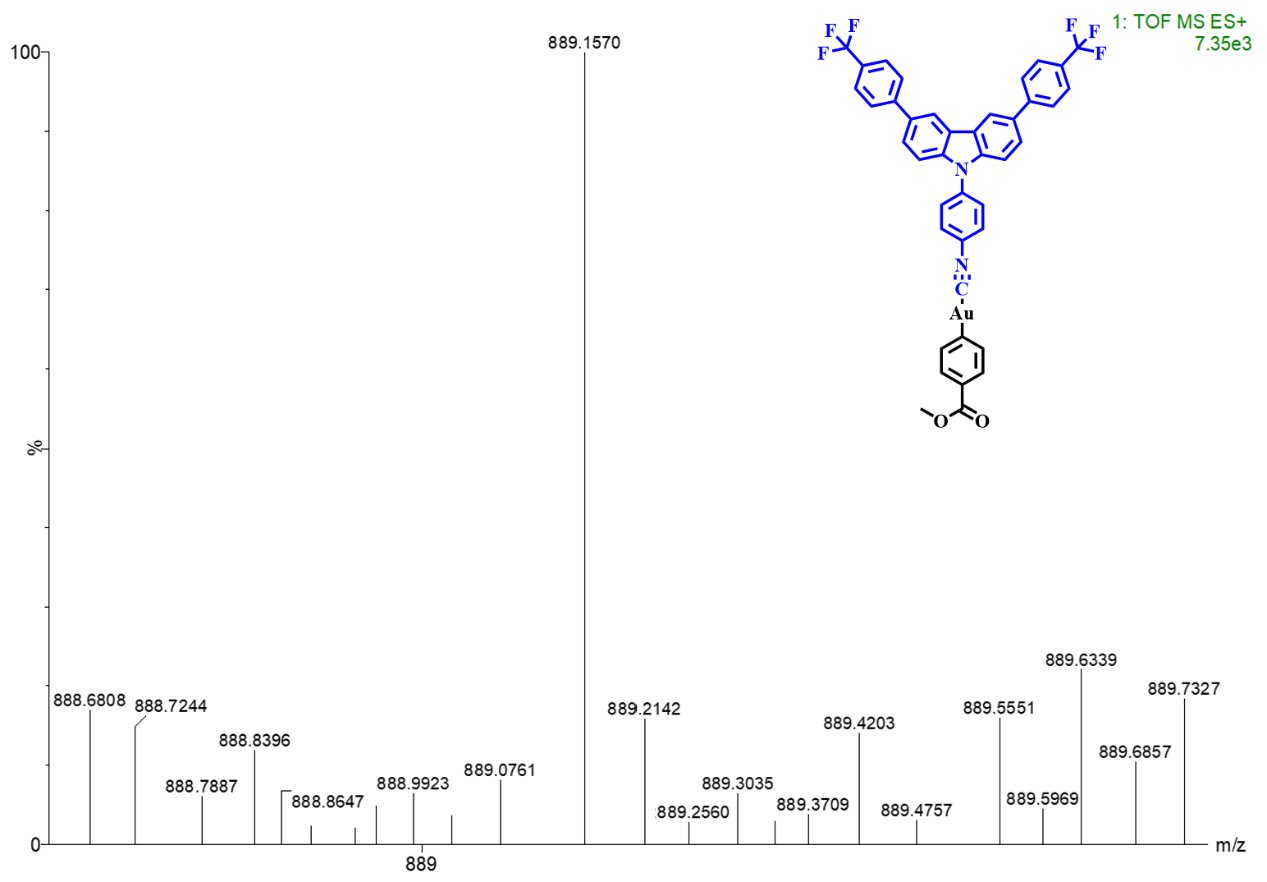
**

**FIGURE. S9 |** Mass spectra of complex **1.**


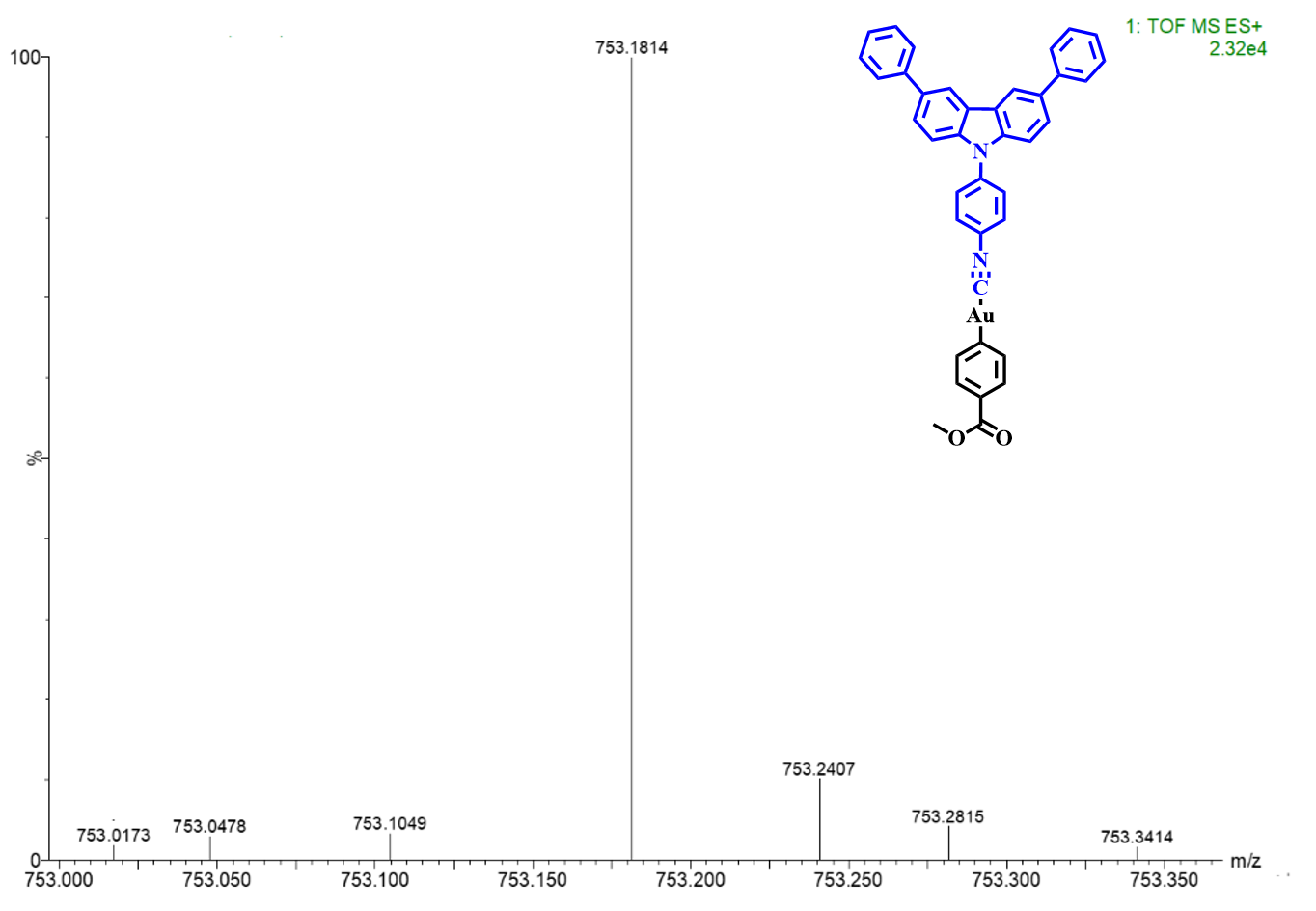


**FIGURE. S10 |** Mass spectra of complex **2**.


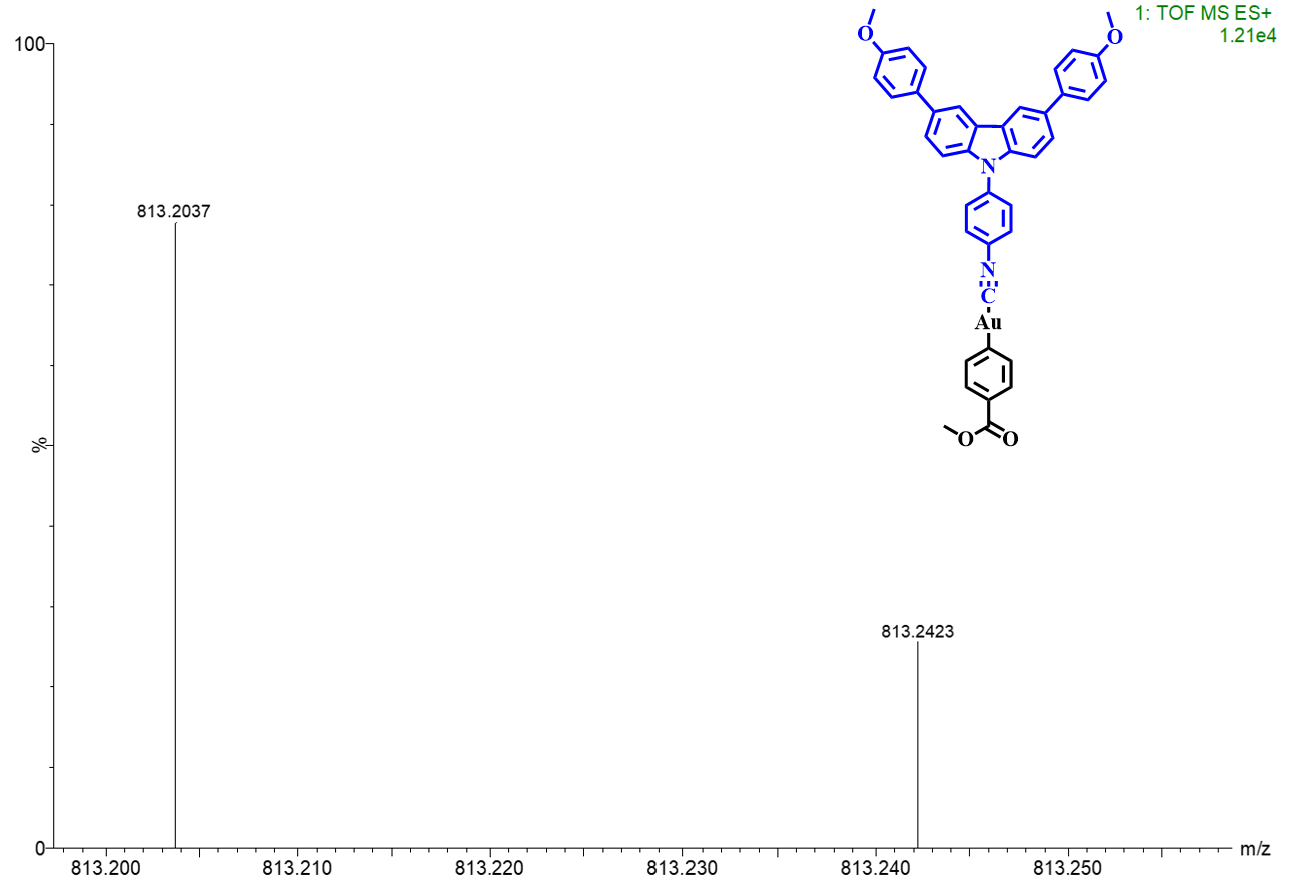


**FIGURE. S11 |** Mass spectra of complex **3**.
